# Epigenetics Meets CAR-T-Cell Therapy to Fight Cancer

**DOI:** 10.3390/cancers16101941

**Published:** 2024-05-20

**Authors:** Simeon Santourlidis, Marcos J. Araúzo-Bravo, Lars Erichsen, Marcelo L. Bendhack

**Affiliations:** 1 Institute of Transplantation Diagnostics and Cell Therapeutics, Medical Faculty, Heinrich-Heine University Duesseldorf, Moorenstr. 5, 40225 Duesseldorf, Germany; lars.erichsen@uni-mainz.de; 2 Group of Computational Biology and Systems Biomedicine, Biodonostia Health Research Institute, 20014 San Sebastián, Spain; marcos.arauzo@biodonostia.org; 3 IKERBASQUE, Basque Foundation for Science, 48013 Bilbao, Spain; 4 Department of Cell Biology and Histology, Faculty of Medicine and Nursing, University of Basque Country (UPV/EHU), 48940 Leioa, Spain; 5 Institute of Developmental Biology and Neurobiology, Johannes Gutenberg University, 55099 Mainz, Germany; 6 Department of Urology, Red Cross University Hospital, Positivo University, Rua Mauá 1111, Curitiba 80030-200, Brazil; marcelo@uro-onco.net

**Keywords:** Car-T-cell therapy, hypomethylation, Car-T-cell targets, epigenetics

## Abstract

**Simple Summary:**

Cancer treatment could be revolutionized by using particular CAR-T-cell therapies for all solid tumors. Finding appropriate CAR-T-cell antigens for every tumor entity would be necessary for this though. Our findings provide new insight into the possibility of employing CAR-T-cell therapy to treat nearly all cancers, as genome-wide screening following consistent occurring DNA hypomethylations may uncover novel antigens for every cancer entity.

**Abstract:**

Based on the impressive success of Car-T-cell therapy in the treatment of hematological malignancies, a broad application for solid tumors also appears promising. However, some important hurdles need to be overcome. One of these is certainly the identification of specific target antigens on cancer cells. Hypomethylation is a characteristic epigenetic aberration in many tumor entities. Genome-wide screenings for consistent DNA hypomethylations in tumors enable the identification of aberrantly upregulated transcripts, which might result in cell surface proteins. Thus, this approach provides a new perspective for the discovery of potential new Car-T-cell target antigens for almost every tumor entity. First, we focus on this approach as a possible treatment for prostate cancer.

## 1. Introduction

Historically, patients with refractory and/or relapsed (R/R) B-cell lymphomas had poor outcomes and bad prognoses following chemoimmunotherapy [1]. Meanwhile, many patients with previously incurable hematological malignancies have been cured by engineering the patient’s own T cells to selectively attack and remove tumor cells [2]. For this CAR-T-cell therapy strategy, a patient’s T cells are collected and prepared ex vivo by a genetic modification to express a synthetic receptor with binding affinity to a specific tumor antigen. Afterwards, they are re-infused back into the patient to exert their curing function against tumor cells presenting the appropriate antigen on their surface. Remarkably, complete response rates of 40–54%, 67% and 69–74% have been reported in patients with refractory and/or relapsed aggressive B-cell lymphomas, those with mantle cell lymphomas and those with indolent B-cell lymphomas [1]. In addition, astonishing successes have been demonstrated in patients with (R/R) multiple myeloma by applying CAR-T cells that target the B-cell maturation antigen (BCMA). In these trials, overall response rates of 73–98% have been shown [1]. This led to the currently fast expanding and rapid approvals and applications of chimeric antigen receptor (CAR) T-cell therapies [1]. Over eleven hundred clinical trials have been launched globally, according to https://clinicaltrials.gov/, accessed on 20 February 2024.

A substantial risk of developing short-term acute undesirable effects after CAR-T-cell therapy exists. Among them, the most important are cytokine-release and neurotoxicity syndromes. Both generally occur within the first month of treatment [1]. Among the most often observed long-term adverse effects are hypogammaglobulinemia, B-cell depletion, cytopenias and infections [1]. It is thought that, in part, these events are due to the fact that the target antigens CD19 and BCMA are also expressed on non-malignant B cells and non-malignant plasma cells, respectively. Thus, these healthy and functionally valuable cells are becoming collateral targets of the engineered therapeutic T-cell agents.

However, due to this extraordinary successful proof of principle on hematological malignancies, it is currently being extensively investigated to allow us to extend this therapy option to solid tumors. To these belong, for example, lung, glioblastoma, glioma, neuroblastoma, head and neck, sarcoma, breast, ovarian, kidney, renal, bladder and prostate cancer [3].

In one recent study, the application of a chimeric antigen receptor (CAR)-T-cell immunotherapy against prostate cancer (PCa) is reported [4]. The patient-derived CD8+ T cells were designed to target prostate-specific membrane antigens (PSMAs) and injected into PSMA-expressing prostate tumors in mice. As a result, the T cells infiltrated the tumors and caused apoptosis and the suppression of tumor growth [4]. Such a result distinguishes this new strategy as a potential new therapy approach for men with metastatic PCa [4]. Of course, CAR-T cell therapy can be combined with other treatment strategies for PCa, e.g., androgen deprivation therapy, radiotherapy or chemotherapy [5]. Notably, CAR-T-cell therapy for PCa could be applied as a focal therapy. To this end, new supporting three-dimensional scaffolds have been developed that improve the delivery, expansion, and activation of CAR-T cells to enhance their therapeutic effects on solid tumors [6].

CAR-T-cell therapy in solid tumors is rapidly developing, with hundreds of clinical trials in progress. Here, it raises unique clinical challenges; among the most important are the difficulty in trafficking cells to tumors and problems with finding specific antigen targets [3]. This last point about finding suitable specific antigen targets is currently highlighted by many groups working in this field. Hence, a new strategy leading to a plethora of specific antigen targets for every solid tumor will revolutionize this therapy approach that has promising potential. This would, e.g., counteract the antigen target-related downregulation of cancer cells during therapy, the heterogeneity of antigen target presentation and unwished side effects by largely avoiding the use of target antigens also presented by other functionally valuable, healthy cells. Of overriding importance are new approaches to improve CAR-T-cell specificity and safety in order to take advantage of its whole therapy potential in solid tumors.

Meanwhile, we see that current research and new progress in the field of epigenetics is likely to make a decisive contribution to one of the necessary and most important steps involved in making this therapy applicable to all cancer entities: to identify, based on screenings for consistent hypomethylations, tumor-specific, unique and potent target antigens, promising to be largely free of side effects. DNA hypomethylation events occur in many cancers, e.g., cervical, ovarian, lung, colon, breast, bladder and prostate cancer, and represent a ubiquitous feature of carcinogenesis [7]. The process of hypomethylation is often observed during the early stages of tumorigenesis, and it becomes more advanced when the tumor progresses or the degree of malignancy increases [7]. The hypomethylation of gene promoters correlates with transcriptional accessibility and competence for expression, where the spread of hypermethylation via regulatory DNA elements is associated with chromatin condensation and gene silencing [8,9]. The ability to influence expression as a function of methylation is not the same for every CpG within a CpG island of a certain gene. Some CpG dinucleotide positions, whether they are methylated or unmethylated, have a stronger impact on gene silencing or activation, respectively, than others [10]. The effects can be mediated through the interference of the methylated or unmethylated state of a certain CpG with regulators of transcription [11]. Many genes have been introduced by others to be hypomethylated in various cancer types [12]. Their epigenetic deregulation, resulting in expression or increased protein function, is involved, e.g., in the support of cell proliferation, migration and invasion [12].

It is proposed here that such hypomethylations concealing the potential to lead to new, potent CAR-T-cell targets in cancer should preferentially be associated with such genes that have tissue-specific expressions with a confined functional relevance inside their natural habitats. Hence, if they were temporarily compromised by CAR-T cells, this would at most lead to negligible functional loss within the patient. For instance, this would be expected when they are temporarily expressed, e.g., in spermatid development or other already-overcome developmental stages, or, for example, in a small specific lymphocyte population of limited functional relevance. Due to these features, they would presumably have a reduced potential to constitute dangerous collateral targets. Thus, their impairment by CAR-T cells is not expected to result in a severe functional failure, endangering the patient.

## 2. Materials and Methods

The clinical samples that have been received from three clinical centers in Europe, the DNA methylation microarray scanning procedure and the analyses of the data are described in our recent publication [13]. In brief, we used hematoxylin- and eosin-stained sections from formalin-fixed paraffin-embedded tissue specimens from prostatectomies and biopsies of pT2, pT3 and pT4 tumors. They were pathologically reviewed for the identification of tumor content (>90%), tumor/adjacent healthy tissue (50/50%) and benign prostatic hyperplasia (BPH) (>90%). A trained pathologist marked the targeted areas. Then, 5 μm slices were cut, microdissected and transferred into reaction tubes for further epigenetic analyses [13]. By repeating all methodological steps with 15 PCA samples and 5 reference samples, as specified [13], blinded internal validation was applied. The director of the Heinrich-Heine-University’s Coordinating Center for Clinical Investigations, along with another colleague, was present during the deblinding process. The outcome was that our computational biology method was able to correctly identify 14 out of 15 PCa cases and 4 out of 5 BPH samples based on the redetected same CpG island hypomethylations by comparing the differentially methylated CpG islands of the new data set to those of the first data sets. The approach mislabeled a tumor sample as a BPH sample in one instance. Therefore, rigorous reproducibility within the same sample cohort was established through the internal validation. By contrasting the outcomes of the external sample cohort from Clinical Center No. 3 with those of the earlier analyses, a blinded external validation was carried out to verify repeatability [13]. The Coordination Center of Clinical Studies (KKS) in Düsseldorf anonymously received the array data from these prostate gland tissue DNA samples (Clinical Center No. 3) [13]. They used this information to blindly compare the differentially methylated regions based on 60 bp probes with those based on whole gene promoter-associated CpG islands from the PCa samples supplied by Clinical Center No. 2. [13]. Forty of the one hundred differentially methylated genes were identified.

## 3. Results

**Figure 1:**
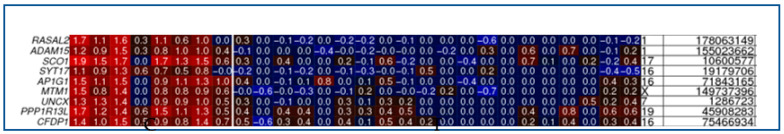
A small excerpt of one DNA methylation heat map from our publication. Araúzo-Bravo et al. [13] is presented, which reveals differential methylations between PCa-adjacent (C) and PCa tissue samples (T).

In our recent publication, we presented a new approach in the field of epigenetic research that provides a possible solution in this direction, as a start, for treating PCa [13]. In three patient cohorts from three independent European clinical centers, we identified, after internal and external validation, consistently occurring hypomethylations. They were named tumor-cell-specific differential methylated CpG dinucleotide signatures (TUMSs). For instance, we found them to be consistently occurring in 20 PCa tissue samples but not in the tumor-adjacent tissue (Figure 1). In this publication, we hypothesize that these PCa-specific hypomethylations arise due to grave methyl group-metabolism disturbances, and we make the observation that many of them persist in loci that are known to be functionally not relevant for PCa [13]. Obviously, the PCa cells endure these differential methylated regions (DMRs), which, in part, result in the aberrant upregulation of genes that will not adopt a role for PCa cell function and phenotype. For those, we chose the term “pleiotropic” expressed genes.

This excerpt exemplarily displays nine differentially methylated, 60-nucleotide-long CpG-rich probes, each associated with the 5′ regulatory regions of the corresponding, named gene on the left. In total, we identified 220 hypomethylated genetic regions in PCa via this approach [13]. Their methylation statuses in eight tissue samples from tumor-adjacent, healthy gland tissue (C) are compared to the ones in 20 PCa tumor tissue samples (T). The chromosomal location of these segments is displayed on the right-hand side (chr, start), and they are represented by colored rectangles. The more red the color, the higher the methylation. Hence, CpG-rich segments that are light blue are the most hypomethylated. The higher the number within a colored rectangle, the higher the methylation [13].

In our study, we found dozens of genes with cell-type-specific expression and a specialized function to be hypomethylated within their CpG island or “shore” at their 5′ region in PCa [13]. Among them, for example, the following genes have been presented: Achaete-Scute Family BHLH Transcription Factor 2 (*ASCL2*), Acrosin-Binding Protein (*ACRBP*), Complement C1r (*C1R*), Complement C3 (*C3*), Fetuin B (*FETUB*), Heme Oxygenase 1 (*HMOX1*), HYDIN Axonemal Central Pair Apparatus Protein (*HYDIN*), Myosin Heavy Chain 4 (*MYH4*), Olfactory Receptor Family 10 Subfamily H Member 4 (*OR10H4*), Protein C Receptor (*PROCR*), Syncollin (*SYCN*) and Killer-Cell Immunoglobulin-Like Receptor with two Ig domains and long cytoplasmic tail 3 (*KIR2DL3*) [13]. According to the Cancer Genome Atlas Program (TCGA) data, *ACRBP*, *FETUB*, *HMOX1* and *KIR2DL3* are overexpressed in PCa [13,14]. Hypomethylated *KIR2DL3* gene promoters and KIR2DL3 expression occur mainly in NK cells. These cells use a unique clonotypic expression mode for *KIR* genes. They regulate them by applying differential DNA methylation and chromatin organization on small CpG islands within the 5′-regulatory regions of *KIR* genes [15,16,17]. It has been demonstrated that hypomethylation is a sufficient criterion to induce KIR receptor expression, including KIR2DL3, on the cell surface [15]. Of note, various KIR receptors have been found to be expressed on non-small-cell lung cancer (NSCLC) tumor cells [18]. It is unknown if KIR receptors are present on the surfaces of PCa cells.

## 4. Discussion

Based on in-house-developed computational biology analyses, we identified short, differential methylated CpG-rich DNA fragments that are consistently present in all PCa specimens (Figure 1) [13]. The same approach is applicable for all solid tumors. For instance, we have finished comparable analyses for urothelial cancer. We pursue the same approach for breast, lung and colon cancers. These consistent TUMSs of a certain tumor entity would then be scrutinized for an aberrant expression of the corresponding genes and whether these fulfil the mentioned requirements to potentially serve as target antigens for a potent CAR-T-cell therapy. For that, they should be expressed on the surfaces, e.g., of PCa cells. For PCa, we representatively present in Table 1 a few candidates identified to be hypomethylated in our patient cohorts, and their expression is enhanced in PCa according to the TCGA data or other published studies. According to this paradigmatic excerpt in Table 1, our approaches revealed many other hypomethylations associated with upregulated genes, encoding for membrane proteins, with the potential to be aberrantly expressed in PCa or urothelial cancer. Simultaneously, they are known for their normal expression and highly specialized function in specific tissues and/or already successfully overcoming developmental stages. These features ensure that a stress produced via CAR-T-cell therapy, especially when focally applied, may not result in unacceptable collateral damage to healthy tissues.

According to this epigenetic approach and, of course, performing follow-up subsequent immunohistochemical analyses to detect the relevant proteins on the surfaces of PCa cells, the cancer-cell-specific potential CAR-T cell targets would be identified. Then, they would comprise antigen combinations to serve in CAR-T-cell therapies, as mentioned above. Importantly, antigen escape has been identified as a main mechanism responsible for disease relapse after CAR-T-cell therapy. This has been confirmed in 20–28% of patients with B-cell lymphoma, in lower incidences in multiple myeloma patients and in 16–68% of patients with B-ALL [1]. This is considered to be the most important factor affecting the permanence of response to CAR-T-cell therapy. In an attempt to overcome this problem, dual antigen targeting appears promising and is being investigated in several clinical trials [1]. Therefore, we pursue dual and triple combinations for all solid tumors, and our epigenetic approach is promising for identifying the requested candidates in sufficient numbers for combinatorial trials. Once we have identified dual and triple combinations, including the successful proof of their presence on the cancer cell’s surface, we will proceed in collaboration with other researchers to design appropriate CAR-T-cell constructs. Our institute has many years of experience in the isolation and efficient transfection of lymphocytes, including NK and T cells [15,16,19]. 

Prof. Marcelo Bendhack is one of the pioneers of high-intensity focused ultrasound (HIFU) therapy for PCa worldwide. He first started in 1994 at the Investigational Center of the University Hospital (UKD) of Heinrich-Heine-University, Duesseldorf, Germany, and since 2011, he has continued the appliance of HIFU in his clinic (2 centers), which is related to the Department of Urology, University Hospital, Positivo University, Curitiba, Brazil. He has successfully treated, since then, more than 850 PCa Patients. Before HIFU became his main interest, he performed around 800 radical prostatectomies. The oncological outcomes of HIFU are very satisfying, as documented in the most important international publications on that subject [20]. Among those patients, there were a few cases of relapse or, better defined, a second occurrence (second focus of malignant disease). They were usually treated via a second course of HIFU therapy or one course of radical salvage surgery, respectively, considering the patient’s clinical scenarios and preferences. Based on his comprehensive experiences in the clinic, Prof. Bendhack underlines the urgent requirement of the development of convincing markers for interrogating field effects and/or evaluating negative biopsy regions (typically a six-region model) as possible foci of future second occurrence within the prostate, and he pushes their development.

Firstly, Slaughter et al. [21] introduced the concept of field effect in cancer, also known as field defect or field cancerization, based, among other things, on their observations of microscopic, hyperplastic abnormalities of contiguous, benign tissue. They considered it as an important factor in the recurrence of cancer after therapy [22]. Today, using modern molecular biology techniques, molecular abnormalities have been revealed in diverse tissues that appear histologically normal. These comprises the head and neck, lung, colon and rectum, breast, stomach, prostate and bladder. These observations have contributed to establishing the field effect as a crucial mechanism involved in the multicentricity of cancer [22]. However, the underlying mechanisms of the field effect in cancer are not fully understood, but it is thought, based on growing molecular evidence, that genetically altered cells and alterations in DNA methylation patterns play a central role here [22].

In accordance with this, PCa has been shown to be almost always multi-focal [23] with cytomorphologic, genetic, epigenetic and gene/protein expression abnormalities noted in the histologically benign tissue adjacent to PCa [24]. For example, Mehrota et al. used 159 biopsy cores from 37 prostatectomy samples and detected an epigenetic field effect for the genes *APC*, *RARb2* and *RASSF1A* up to 3 mm from the malignant core in three prostatectomy samples [24].

For the treatment of localized prostate cancer, focal therapy (FT), a group of minimally invasive methods, e.g., high-intensity focused ultrasound (HIFU), Focal Cryotherapy, Irreversible Electroporation (IRE), focal brachytherapy, Focal Laser Ablation (FLA), etc., shows promise as an alternative to whole-gland procedures. [25]. Due to its excellent results in terms of safety and functional outcomes, an expanding distribution of FT is likely. However, we must be aware that the oncological effectiveness of FT in comparison to the standard of care is still under investigation [26].

Prostate cancer has a heterogeneous clinical outcome and is a multifactorial, complex disease. Which malignant focus has the greatest chance of developing into a more serious and possibly fatal illness and which focus has the potential to initiate recurrence after therapy remains unknown. Despite this difficulty, single-sample testing is frequently used in research and clinical practice [27]. However, the truth is that spatial heterogeneity exists both between the malignant foci (interfocal) and within foci (intrafocal) in PCa [27], which is thought to contribute to distinct prognoses and treatment responses after FT.

At this point, a crucial clinical need appears that requires potent biomarkers to define the most relevant primary malignant foci and be informative for the whole organ in order to decide for or against whole-organ treatment, such as radical prostatectomy [27]. Similarly, biomarkers with established utility for particular foci should direct the decision to treat only these areas. Here, TUMSs, which are consistently present in PCa, have the potential to provide the urgently required relief to further improve oncological outcomes.

A further application of TUMSs would be their usage in liquid biopsies. Here, bodily fluids, e.g., blood, urine, etc., are removed, and the analysis of cell-free DNA (cfDNA) is conducted. This method is patient-friendly, since it is minimally invasive at best and cost-effective. It has been documented that in healthy donors, cfDNA is released via the cellular processes of apoptosis, necrosis and secretion. Its concentration does not exceed 5–10 ng/mL [28]. It is shown that the main origins of this DNA fraction are white blood cells (55%), erythrocyte progenitors (30%), vascular endothelial cells (10%) and hepatocytes (1%) [29]. In addition, it has been demonstrated that the plasma of older people shows significantly higher levels of total cfDNA [29]. In cancer, additional cfDNA is released by the apoptosis or necrosis of dying tumor cells or shed by viable tumor cells [30]. Hence, the total cfDNA concentration may increase by up to 50-fold compared to healthy persons. This depends on the type of cancer and the burden of disease [28]. Interestingly, total cfDNA levels decrease after therapy or surgery for cancer [30]. We conclude that TUMSs would be applicable to detect and clinically exploit this cancer cell DNA fraction on the basis of distinct cancer-cell-specific DNA hypomethylation profiles.

If we consider the total number of patients with recurrence after surgery, radiation therapy, hormonal therapy, immunochemotherapy or even initial metastatic disease, this new therapy approach with CAR-T cells is certainly of the highest interest for many clinical scenarios. Improvements in tumor marker development and promising therapy approaches like CAR-T cells are urgently needed and are being vehemently pursued and sported.

**Table 1:** This is a small excerpt of many hypomethylated genes in PCa identified by Araúzo-Bravo et al. [13]. It places candidates in the context of other studies’ evidence about how they manifest in cancer. They will be further investigated to determine whether they have the properties to serve as antigens for CAR-T-cell therapy in PCa. This epigenetic screening strategy is a platform approach used to identify potential CAR-T-cell antigens for all solid tumors [13].

Gene Symbol/ Name/ Gene ID/ (Chromosomal Location)	Function GeneCards (www.genecards.org) [31]	Expression Human Protein Atlas proteinatlas.org [32]	The Cancer Genome Atlas Expression (TCGA) in PCa [14]	References in PubMed https://pubmed.ncbi.nlm.nih.gov/
***KIR2DL3***/ Killer-cell immunoglobulin-like receptor 2DL3/ 3804/ (19q13.42)	Killer-cell immunoglobulin-like receptors (KIRs) are transmembrane glycoproteins with an important role in the regulation of the immune response.	Expressed by NK cells and subsets of T cells.	1.38	Hypomethylation, as reported by us [13], is sufficient for expression in NK cells [15]. KIR receptor expression has been demonstrated on NSCLC tumor cells [18].
***SLC27a4***/ solute carrier family 27 member 4/ 10999/ (9q34.11)	This protein plays a role in the translocation of long-chain fatty acids in the plasma membrane.	Expressed on mature enterocytes in the small intestine. Membrane, intracellular (different isoforms).	1.79	This protein is overexpressed in 21 types of human cancer, e.g., ovarian cancer, hepatocellular carcinoma and breast cancer [33].
***SLC52a2***/ solute carrier family 52 member 2/ 79581/ (8q24.3/)	This membrane protein belongs to the riboflavin transporter family mediating the uptake of the water-soluble vitamin B2/ riboflavin.	Cytoplasmic expression in most tissues. Membrane, intracellular (different isoforms).	1.78	SLC52A2 is highly expressed in almost all tumors. Immunohistochemical results have confirmed this in hepatocellular, gastric, colon and rectal cancers [34].
***ADAM15****/* *ADAM metallopeptidase domain 15/* *8751/* (1q21.3)	This type I transmembrane glycoprotein interacts with the integrin beta chain beta 3. It is thought that it functions in cell–cell adhesion, as well as in cellular signaling.	Cytoplasmic expression in most tissues. Membrane and intracellular (different isoforms).	1.32	ADAM15 is highly expressed in PCa metastasis and interacts with vascular endothelium [35]. It has been shown that negative (87.7%), weak (3.7%), moderate (5.6%) and strong (3.0%) ADAM15 staining was found in 9826 prostate tumors. Strong expression has been linked to high Gleason grade, advanced pathological tumor stage and positive nodal stage [36].
***ABCA7**/* ATP binding cassette subfamily A member 7/ 10347/ (19p13.3)	The function of this protein has not been elucidated yet; due to its expression pattern, it has been suggested that it might play a role in lipid homeostasis in cells of the immune system.	Lymphoid tissue and bone marrow—innate immune response. Plasma membrane. It is additionally localized to the cell Junctions.	0.987	The ABC transporter ABCA7 plays a role in lipid transport processes and cholesterol homeostasis. In a variety of cancer types, it is aberrantly expressed. This is also the case in breast cancer [37].
***BCAM****/* basal cell adhesion molecule (Lutheran blood group)/ 4059/ (19q13.32)	This gene encodes a receptor for the extracellular matrix protein, laminin. It is thought to play a role in epithelial cell cancer.	Membranous expression in basal membranes and endothelial cells.	2.0	CD239 promotes the migration of lung carcinoma cells on laminin-511. The over-expression of CD239 is observed in ovarian carcinoma, skin cancer and hepatocellular carcinoma. CD239 is strongly expressed in a subset of breast cancer tissues and cells [38].
***PLEC**/*plectin/5339/8q24.3	This protein interlinks different elements of the cytoskeleton and orchestrates dynamic changes in cytoarchitecture and cell shape.	Membranous and cytoplasmic expression in almost all cells.	0.940	Localized and metastatic human PCa shows high levels of plectin. Plectin knock-down inhibited decreased overall metastatic burden [39]. Plectin has a cancer-specific **mislocalization** on the cell surface. This is involved in its function as a potent oncoprotein [40].
***TNFRSF4***/TNF receptor superfamily member 4/7293/1p36.33	This receptor has been shown to activate NF-kappaB. Evidence is provided that this receptor suppresses apoptosis.	This protein has cytoplasmic expression in spleen, tonsil and lymph node. Membrane, intracellular (different isoforms).	1.38	*TNFRSF4* provides co-stimulatory functions of T cells during infection. It is transiently and predominantly expressed by both human CD4+ and CD8+ T cells [41].
***KCNS3***/potassium voltage-gated channel modifier subfamily S member 3/3790/2p24.2	These voltage-gated potassium channels control the resting membrane potential and the shape and frequency of action potentials.	Membranous and cytoplasmic expression in most tissues.	1.58	KCNS3 has been identified as part of a prognostic signature separating high- and low-risk groups in esophageal squamous cell carcinoma (ESCC) patients [42].

## 5. Conclusions

For the successful and broad application of Car-T-cell therapy in solid tumors by largely avoiding side effects, it is desirable to draw on a plethora of possible target antigens. High-resolution screening for consistent hypomethylations in CpG-rich fragments associated with regulatory gene regions provide basic hints about potential upregulated surface proteins that may serve as new, potent Car-T-cell antigens. In addition to this, TUMSs, which are consistently present in cancer, hold potential for high-resolution, personalized diagnosis and prognosis, both from tissues and minimally invasive circulating cfDNA.

## Data Availability

The data presented in this study are available on request due to privacy/ethical restrictions.
